# Health Care Leaders’ Perspectives on the Maryland All-Payer Model

**DOI:** 10.1001/jamahealthforum.2021.4920

**Published:** 2022-02-04

**Authors:** Austin S. Kilaru, Christina R. Crider, Joshua Chiang, Elisabeth Fassas, Katherine J. Sapra

**Affiliations:** 1 Center for Emergency Care Policy and Research, Department of Emergency Medicine, Perelman School of Medicine, University of Pennsylvania, Philadelphia; 2 Leonard Davis Institute of Health Economics, University of Pennsylvania, Philadelphia; 3 Center for Medicare and Medicaid Innovation, Centers for Medicare & Medicaid Services, Baltimore, Maryland; 4 currently a medical student at Perelman School of Medicine, University of Pennsylvania, Philadelpia; 5 currently a medical student at University of Maryland School of Medicine, Baltimore

## Abstract

**Importance:**

Since 2014, all hospitals in Maryland have operated under an all-payer global budget system. Hospital global budgets have gained renewed attention as a strategy for constraining cost growth, improving patient outcomes, and preserving health care access in rural and underserved communities. Lessons from the implementation of the Maryland All-Payer Model (MDAPM) may have implications for policy makers, payers, and hospitals in other settings seeking to adopt global budgets or other value-based payment models.

**Objective:**

To examine perspectives on the implementation of the MDAPM among health care leaders who participated in its design and execution.

**Design, Setting, and Participants:**

This qualitative study with semistructured telephone interviews was conducted from November 1, 2019, to February 11, 2020. The purposive sample of Maryland health care leaders represents diverse stakeholder groups, including hospitals, state government and regulatory agencies, the federal government, and payers.

**Main Outcomes and Measures:**

Key high-level themes were extracted from interviews using qualitative content analysis, with barriers and facilitators to implementation specified within each theme.

**Results:**

A total of 20 interviews were conducted with hospital leaders (n = 6), state regulators (n = 4), federal regulators (n = 4), payer representatives (n = 3), and state leaders (n = 3). Key themes were labeled as (1) expectations (setting bold yet achievable goals), (2) autonomy (allowing hospitals to follow individual strategies within MDAPM parameters), (3) communication (encouraging early and ongoing communication between stakeholders), (4) actionable data (sharing useful hospital and patient-level data between stakeholders), (5) global budget calibration (anticipating technical challenges when negotiating budgets for individual hospitals), and (6) shared commitment to change (harnessing collective motivation for system change). Together, these themes suggest that implementing the payment model followed an evolving and collaborative process that requires stakeholder communication, data to guide decisions, and commitment to operating within the new payment system.

**Conclusions and Relevance:**

The implementation of hospital global budgets in the state of Maryland offers generalizable lessons that can inform the evolution and expansion of this approach to value-based payment in other states and settings.

## Key Points

**Question:**

What lessons can be extrapolated from the implementation of hospital global budgets in the Maryland All-Payer Model that apply to other communities, payers, and hospitals seeking to adopt value-based payment systems?

**Findings:**

This qualitative study of Maryland health care leaders revealed key themes regarding hospital global budgets, including setting achievable expectations for health care transformation, protecting hospital autonomy, ensuring close communication between stakeholders, using actionable data to inform decisions, carefully calibrating budgets, and harnessing a shared commitment to change.

**Meaning:**

The findings of this study suggest that the experience of implementing hospital global budgets in Maryland can inform the development of future payment models that seek to constrain cost growth, improve patient outcomes, and preserve access to health care.

## Teaser

This qualitative study examines perspectives on the Maryland All-Payer Model among health care leaders who participated in its design and implementation and identifies themes that may be generalized to other settings.

## Introduction

In 2014, Maryland implemented global budgets for all acute care hospitals.^1,2^ Developed as a model test by the Centers for Medicare & Medicaid Services (CMS), Center for Medicare and Medicaid Innovation, and Maryland’s Health Services Cost Review Commission (HSCRC), the Maryland All-Payer Model (MDAPM) specified annual targets for growth in Medicare and all-payer hospital expenditures.^3^ The global budget, consisting of hospital inpatient and outpatient revenue, was the method to achieve those targets.^4^ The state had previously used a rate-setting system that regulated hospital prices for all payers, operating under an exemption from the Medicare Inpatient and Outpatient Prospective Payment Systems.^5^ The agreement to establish the MDAPM allowed the state to continue this exemption and build on 4 years of experience in setting global budgets for 10 rural hospitals.^6^

As an alternative to fee-for-service payment, hospital global budgets represent an opportunity to constrain growth in cost while strengthening quality incentives.^1,4^ In this system, revenue no longer depends on the volume of admissions, emergency department visits, and outpatient services. Hospitals may be motivated to reduce avoidable use, whether by preventing readmissions, reducing iatrogenic complications, or, further upstream, addressing population health.^5,6,7,8,9,10,11^ This approach can incorporate safeguards to ensure that patients receive appropriate care while avoiding excessive financial risk to hospitals.^4^

Global budgets also offer a strategy to stabilize hospital finances.^12^ This system may particularly benefit vulnerable rural hospitals, allowing them to address community health needs rather than compete for volume.^13,14,15^ The Pennsylvania Rural Health Model, also developed by the Center for Medicare and Medicaid Innovation, currently tests this approach.^16,17^ The Community Health Access and Rural Transformation Model will further extend the reach of hospital global budgets into rural communities across the US in 2023.^18^ The COVID-19 pandemic highlighted the susceptibility of hospitals to losses in volume—and the need to prevent closures.^14,19,20,21^

One study estimated that the MDAPM resulted in total savings of $975 million to Medicare for 5 years, although there were no overall savings for commercial plan members because increased professional spending partially offset hospital savings.^7^ However, many questions remain about the impacts of the MDAPM, including its effect on population health outcomes, comparative effectiveness to other alternative payment models, and generalizability beyond Maryland.^7,22,23,24^ Lessons from the MDAPM and its progression to the current Maryland Total Cost of Care Model have important implications for the future of value-based payment.^25^ Implementation of this large-scale transformation, decisions that facilitated that transformation, and the challenges encountered may be of particular salience to hospitals, insurers, and state governments outside of Maryland who may be interested in the hospital global budget approach.

In this study, we examined perspectives on the MDAPM among health care leaders in Maryland who participated in its design and implementation. The goal was to extract high-level themes to identify lessons that may be generalized to other settings. Rather than focus on technical MDAPM details or specific hospital strategies for operating within a global budget system, which have been described previously,^3,26^ this study examined facilitators and barriers to implementing the overall model.

## Methods

In this qualitative study, we conducted semistructured interviews with health care leaders. This study followed the Consolidated Criteria for Reporting Qualitative Research (COREQ) reporting guideline and was approved by the institutional review board at the University of Pennsylvania. Verbal informed consent was obtained from participants with permission to publish deidentified quotations.

### Study Participants

To identify study participants, we used purposive sampling to obtain representation across different stakeholder groups involved in the design and implementation of the MDAPM. First, we mapped stakeholder categories, including Maryland state government, the federal government, hospitals and health care organizations, and payers (eFigure in the Supplement). For this study, we did not include patients or patient representative groups. In discussion with the Center for Medicare and Medicaid Innovation and HSCRC, we identified key informants within each category and recruited them for participation. Key informants were also queried to recommend additional participants. All participants met prespecified criteria that included having (1) a health care leadership role, (2) direct involvement in developing the MDAPM or ongoing contributions to the Maryland health care system, and (3) detailed knowledge of the MDAPM.

### Data Collection

The interview guide was developed using the Consolidated Framework for Implementation Research (CFIR).^27^ The CFIR offers an evidence-based structure to organize potential influences (constructs) on the implementation of a program or intervention. The goal of the interviews was to elicit perspectives and experiences with the MDAPM from which we could extract high-level themes on the design, adoption, and execution of the model, with a focus on facilitators and barriers to implementation. We divided the interview questions (eMethods 1 in the Supplement) according to 5 CFIR domains: (1) readiness for implementation, (2) external forces motivating change, (3) features of the model, (4) leaders and organizations, and (5) decisions and actions.^27^ A final question asked participants to make recommendations to health care leaders in other settings who might adopt hospital global budgets. The interview guide was piloted with unaffiliated health care executives. Minor revisions for clarity were made to the interview guide after the third interview, after which no edits were made.

Telephone interviews were conducted from November 1, 2019, to February 11, 2020. A single study author (A.S.K.) conducted all interviews, with mean length of 57 (range, 28-115) minutes. After each set of 3 interviews, we reviewed transcripts to assess for theme saturation. We defined saturation as the point in which participant responses repeated general themes provided in earlier interviews. All interviews were recorded, transcribed, and entered into NVivo software, release 1.2 (QSR International).

### Data Analysis

To analyze participant responses, we used qualitative content analysis.^28,29^ First, we created a preliminary codebook consisting of 4 general codes that were defined a priori: barriers to implementation, facilitators to implementation, outcomes and consequences, and key decisions. Additional thematic codes were defined according to topics that emerged from initial review of the transcripts and study team discussion. These thematic codes are listed in the codebook (eMethods 2 in the Supplement).

Two authors (J.C. and E.F.) systematically coded the interviews. Five transcripts were independently double coded, after which discrepancies in coding were discussed and resolved. After the codebook was refined by clarifying code definitions and removing unnecessary codes, the remaining interviews were coded. Participant responses attributed to general and thematic codes were extracted and analyzed by the study team. Coding matrices summarized how frequently general and thematic codes were applied to the same section of text, and the most frequently overlapping codes assisted in selection and labeling of potential key themes. From these analyses, the study team consolidated key themes and selected representative quotations. Barriers and facilitators to implementation were nested within these high-level interconnected themes.

## Results

We conducted a total of 20 interviews with hospital leaders (n = 6), state regulators (n = 4), federal regulators (n = 4), payer representatives (n = 3), and state leaders (n = 3). We determined that theme saturation had been achieved after 18 interviews but completed 2 additional interviews to ensure representation from all stakeholder groups. Table 1 lists participant categories and affiliations. Table 2 summarizes the 6 high-level themes, along with representative quotations and relevant CFIR constructs. The study team labeled these themes as (1) expectations, (2) autonomy, (3) communication, (4) actionable data, (5) global budget calibration, and (6) shared commitment to change. Table 3 describes barriers and facilitators to implementation of global budgets contained within each theme.

**Table 1.** Interview Participants Listed by Affiliation

Interview No.	Affiliation
1	State of Maryland
2	Maryland Department of Health
3	Maryland HSCRC
4	Hospital leader (hospital system A)
5	Maryland HSCRC
6	CMMI
7	Maryland Hospital Association
8	Maryland HSCRC
9	Maryland Medicaid
10	Hospital leader (hospital system B)
11	Maryland HSCRC
12	Hospital leader (hospital system C)
13	Hospital leader (hospital system D)
14	CMS
15	Chesapeake Regional Information System for Our Patients
16	CMMI
17	Hospital leader (hospital system E)
18	CMMI
19	Commercial payer (payer A)
20	Commercial payer (payer B)

Abbreviations: CMMI, Center for Medicare and Medicaid Innovation; CMS, Centers for Medicare & Medicaid Services; HSCRC, Health Services Cost Review Commission.

**Table 2.** Key Themes With Representative Quotations

Theme	CFIR constructs	Representative quotations
Expectations	Process (planning, execution)	“If we could go to value-based care in one fell swoop instead of 1 inch at a time and do it on an all-payer basis, that was a much better approach of moving to the future.” (interview 5, state regulator)
		“We expect short-term results from long-term strategies. To anticipate really radical changes, 5 years is a relatively short period of time.… If you could somehow incentivize all providers to decrease the cost of care, that’s the ideal because everyone marches in the same direction. But I think we have to be realistic. When you’re talking about a statewide model, you have to be very realistic as to what you really can accomplish and what your expectations are.” (interview 14, federal official)
Autonomy	Intervention characteristics (adaptability)	“The state’s job is not to tell hospitals what to do. It’s more like, ‘Here’s your capitation payment and you make it work.’ Hospitals have actually done a wide variety of things. Now, the wide variety of things could also be a concern. What are the best practices? Maybe we need to define those better.” (interview 8, state regulator)
		“CMMI realized that Maryland needed to be nimble. We wrote the amendment to give the state more flexibility to make changes and modifications to add programs under the framework of the Care Redesign Program.” (interview 7, hospital representative)
Communication	Inner setting (networks and communication)	“There were many short presentations explaining what was going on and the implementation process and how it was going to roll out over a 5-year period. The most important part was with the hospital association and hospital leadership, and then the work groups and also working with smaller groups of stakeholders.” (interview 6, federal regulator)
Actionable data	Inner setting (structural characteristics, available resources)	“We made sure to elevate the importance of data access for providers across the care spectrum. Not just for hospitals, but having other providers be able to access hospital data. Identifying the data that is important for providers to have at their point of care so that they can best care for and manage the health of their patients in a meaningful way.” (interview 3, state regulator)
Global budget calibration	Intervention characteristics (complexity)	“[Hospitals] were already feeling a big pinch and were really worried about how that would go. I think that they felt a degree of confidence that they could work out the details with the HSCRC. In other states, where you don’t have a regulator like that, there would be no confidence, and people are going to be much more suspicious. Whereas in Maryland, I think the hospitals have a degree of comfort with the HSCRC, understand that they had a voice. They didn’t always agree with the HSCRC, but it didn’t feel that alien to them.” (interview 2, state official)
Shared commitment to change	Outer setting (external policies and incentives)	“What really focused everybody’s mind was that we were going to lose the [rate-setting] system. Everybody was nervous about that because it would destabilize health care in Maryland quite a bit. People thought, ‘Well, how can we solve that?’ Some people said, ‘We should just have more reimbursement for fee for service.’ It became clear that CMS was not interested. So, we had to get people around the idea that the only way that we were going to be able to keep our system intact was to do something that was very different and ambitious.” (interview 2, state official)
	Inner setting (implementation climate)	“The typical competition between hospitals is obviously still there, but the willingness to share best practices and learnings with one another can be phenomenal in this state. I’ve been surprised by how many leaders have been open to having us do tours to learn what they’re doing with their care management process.” (interview 12, hospital leader)

Abbreviations: CFIR, Consolidated Framework for Implementation Research; CMMI, Center for Medicare and Medicaid Innovation; CMS, Centers for Medicare & Medicaid Services; HSCRC, Health Services Cost Review Commission.

**Table 3.** Key Barriers and Facilitators for Study Themes, With Representative Quotations

Theme	Representative quotations	
	Facilitators	Barriers
Expectations	Conversion to global budget sets bold expectations for change: “You can’t have a foot on the dock and a foot on the boat, so we just all got in the boat. The global budgets were the game changer.” (interview 7, hospital representative)	Navigating rapid and extensive changes requires a gradual approach: “Change is hard for any organization. Health care delivery is also hard. I would say to start at a basic level and evolve as you go. Not to expect to have a fully built, sophisticated methodology system for governing global budgets right off the bat. It takes stakeholders and others time to feel comfortable with how they can best deliver care under a global budget. Do not be afraid just to grow incrementally, to evolve gradually.” (interview 3, state regulator)
	Achievable goals for quality and cost provide targets for hospitals: “We were supportive of using population health metrics—things like mortality rates, smoking rates—that made sure as the hospital model was running, we were creating pathways for people to start thinking about how this model would affect broader population health.” (interview 6, federal official)	Gradual change may delay care transformation: “You can have really good incentives for population health, but then it’s hard to get people to behave differently if their culture is different. It’s important to have both high and low expectations…. A lot is happening that, no question, wouldn’t be happening except for this change in alignment. But if you have high expectations, then you’d say we really haven’t yet seen a real investment in community prevention.” (interview 2, state official)
Autonomy	Hospitals drive innovation according to the needs of their population: “The all-payer model allowed us to change our focus from filling hospital beds to actually focusing on why these people in our community were being admitted to the hospital and seeing if we could find a way to improve their health, so that they wouldn’t have been admitted.” (interview 13, hospital leader)	Hospitals vary with regard to engagement in care transformation: “Not everybody has changed from a value-based approach to value at the same rate. I don’t think they’re all 100% there. I think it’s a huge shift, and some of them are still trying to figure out how committed they are. I think the [HSCRC] has challenges to make sure that our policies are consistent with that move. I would say that all of us started with recognition that we had some challenges to address, policies to change. It has been an evolutionary process.” (interview 11, state regulator)
	Quality metrics create safeguards to maintain focus on value: “We focused on trying to drive change in 2 areas, principally, 2 areas, and then allowing hospitals to innovate around those 2 areas. The focus area was on readmissions and prevention quality indicators, so we put large penalties in—we put large sticks and carrots with those programs so that people would focus on innovating around those programs.” (interview 5, state regulator)	Improving alignment between hospitals and physicians required additional federal waivers: “The first 3 years had some inertia before we started to develop ways for the hospitals to work with the physicians and get them to a place where they could be aligned. That required [legislation and] fraud and abuse waivers to be put into place.” (interview 19, commercial payer)
Communication	Trusted champions and clear, frequent opportunities for communication facilitated model acceptance: “There was very good, clear communication between CMS and the state, and then the state had a lot of strong leaders who were able to implement those policies—or pass along the requirements of the model in order to be successful in a way that it could be adhered to by everyone.” (interview 20, commercial payer)	Model complexity requires intensive communication, adding to administrative burden: “It can be challenging to have really meaningful dialog in those conversations because it feels like sometimes you never have exactly the right people in the room. Even if you somehow have this magical person who [has clinical and policy expertise], they probably don’t have enough time because you can’t send your chief medical officer out once a week to go drive to Baltimore to spend 2 or 3 hours at a meeting to talk on a policy that may or may not ever make it fully to the submission. I think that’s another challenge.” (interview 12, hospital leader)
Actionable data	Hospital-level and patient-level data must be available and useful to stakeholders at all levels: “The data within CRISP is available within the [electronic health record]. You’re not logging into a separate portal. It’s all right there—showing you care alerts, care teams, other hospital events, and nonhospital events. It gives clinicians more transparency into where else this patient has been.” (interview 15, health information exchange official)	Developing infrastructure and analyzing data require investment and expertise: “[The Model] needs infrastructure to gather data and to make sure that it is viable. For anyone wanting to do this, they cannot underestimate the investment they have to make in a creditable data system and smart people that understand the numbers to run the system from the HSCRC. And hospitals that do well in this system are the ones that are most sophisticated utilizing that data.” (interview 9, state official)
Global budget calibration	Calibrating global budgets required continued negotiation, communication, and willingness to adapt: “When we started with global budgets, we started along this path: everybody got the same inflation increase, and you get a population increase and a quality adjustment. But giving everybody the same inflation adjustment didn’t work because it turned out that some hospitals had oncology programs and other ones didn’t. If a hospital had an oncology program, it actually needed a much larger inflation factor than the hospital who didn’t have one.” (interview 5, state regulator)	Budgets remain challenging to establish, particularly for urban settings as well as hospitals with special circumstances or needs: “Another barrier is that hospitals have to think about paying for recapitalizing a building, or for instance, putting in a new IT system. In the past, hospitals have been able to grow volume that would allow them to raise money for capital, raise money for their IT infrastructure. That has been a challenge.” (interview 3, state regulator)
Shared commitment to change	Stakeholders shared incentives to collaborate and build a new system together: “I don’t think everybody knew exactly what they were getting into in terms of global budgets. I do think they all knew what they were getting away from, which was near the end of that waiver and a transition to [standard Medicare payment systems]. And so I think it was helpful for everybody to keep that in mind as they worked together—also when some of the global budget and all-payer model components were both developed on the fly.” (interview 16, state regulator)	Circumstances specific to Maryland were important drivers of collaboration and change but may not be generalizable to all settings: “I think [critics] point to Maryland’s unique set up of having an independent rate commission. To be fair, that is definitely a component and contributing factor. At the same time, part of what the program was trying to do was to literally move the system away from rate.… There’s no reason you can’t create a global budget for the hospital and negotiate this yourself. The challenge is then you have to get Medicare to agree that they would pay along those same lines.” (interview 20, commercial payer)

Abbreviations: CMS, Centers for Medicare & Medicaid Services; CRISP, Chesapeake Regional Information System for Our Patients; HSCRC, Health Services Cost Review Commission; IT, information technology.

### Expectations

Study participants noted that Maryland and the CMS established high expectations for the MDAPM to transform care delivery and reduce cost. Conversion to the global budget system was perceived as the first and necessary step to realign hospital priorities, committing them to a new value-based paradigm. As one state regulator noted, “The concept of the global budget is the fundamental piece that flipped the incentives for hospitals in the right direction... It was such a monumental change, a sea change, in the way that hospitals thought about raising revenue [interview 3].” However, meeting these high-level goals required additional measures to soften the impact and pace of the change. Participants acknowledged that developing local strategies to operate within the new paradigm could not be done immediately. As one federal regulator observed, “Changing—from business strategies that chase volume to ones that did not—required some time. That is not something that we, I think, could have reasonably expected to happen overnight [interview 16].” One approach to ease this transition was that the HSCRC preserved hospital revenue without immediately lowering budgets if patient volume decreased. Another strategy incorporated into the MDAPM design was the specification of measurable and achievable targets for quality and cost of care, such as reducing hospital 30-day readmission rates to the national average. Participants noted that capping the annual growth rate for hospital revenues to state gross domestic product also provided a transparent target for cost growth.

### Autonomy

Participants observed both benefits and challenges from allowing hospitals to operate autonomously within the global budget. A state regulator noted that “[hospitals] in Maryland wanted to innovate on their own and not be directed by the regulatory body, and in fact, the regulatory body was not large enough to direct innovation [interview 5].” Participants described a diverse range of hospital initiatives to improve value, adapted for local circumstances and organizational priorities, including transitional case management programs and community health initiatives.

However, participants also noted that hospitals varied with regard to engagement in care transformation. Many perceived that costs were reduced not only by improving patient outcomes but also by shifting some hospital-based services to outpatient settings not regulated by the global budget. A hospital representative noted, “When you look at our success under the All-Payer Model, I know that some of that is quality improvement. In particular, our readmissions reductions are very real, and we put things in place that we needed. At the same time…there were things that hospitals were able to do pretty quickly to drive a significant amount of utilization into other settings [interview 7].” Some participants observed that the surplus revenue under the global budget was often not committed to population health improvement but rather distributed by hospitals according to their own priorities.

To avoid unintended consequences of this autonomy, the MDAPM included safeguards to focus hospital priorities on improving quality of care. These safeguards included incentives based on key quality metrics, including unplanned hospital readmissions and rates of iatrogenic complications. Participants noted that these incentives and other aspects of the MDAPM, such as rate corridors that constrained the ability of hospitals to dramatically increase or decrease charges for services, were perceived to help prevent hospitals from eliminating services or withholding care to reduce cost.

After the implementation of the MDAPM, a persistent challenge for hospitals was aligning incentives with physicians who continued to operate under fee-for-service payment structures. Hospitals needed greater autonomy to financially reward physicians who engage in specific care redesign activities that would likely improve the quality and reduce the cost of hospital care. To address this challenge, the CMS and Maryland introduced the Care Redesign Program in 2017, a voluntary program that allowed hospitals to share resources and create appropriate incentives for nonhospital health care professionals and suppliers.

### Communication

Many participants noted that close communication among the CMS, HSCRC, hospitals, and other health care entities was critical to successful MDAPM implementation, both for initial negotiations between the CMS and the state to create the model as well as later navigation of operational details by hospitals. Strategic decisions regarding communication also facilitated acceptance of the MDAPM among hospitals. State officials identified champions within each stakeholder group with influence to persuade their peers and shared a vision for the future model. As one state official noted, “We asked, who were the leaders that would really understand this and be able to work with us?... We didn’t go right to the most suspicious hospital CEO. We needed someone who could convince that person [interview 2].” Later, the HSCRC established work groups to design and provide feedback on various aspects of the MDAPM. Some participants noted that the geographic proximity of all stakeholders in Maryland, including the CMS, facilitated communication. However, a barrier was the intensive communication needed to navigate the complex technical and clinical details involved in the MDAPM, adding to administrative burden for hospitals and requiring frequent engagement with state and federal regulators.

### Actionable Data

Nearly all participants cited the importance of data infrastructure for the design, implementation, and sustainability of the MDAPM. Data on hospital utilization and cost informed development of the model and facilitated acceptance among stakeholders. One state regulator commented, “The one thing that is absolutely critical to what we were able to do is the presence of a lot of data…. We had a tremendous amount of information that we shared openly…and that doesn’t exist in many places [interview 5].” Data sources included Medicare claims, which required special arrangements with the CMS to expedite sharing with the state, as well as quality reports from hospitals. Data also included patient-level information on health care utilization, medical history, and patient care plans extracted from electronic health record systems, which were incorporated into the state health information exchange (Chesapeake Regional Information System for Our Patients). This information exchange was widely perceived as essential for implementation of the MDAPM to monitor hospital volume in real time and identify opportunities to improve value. The development of this system required investment from the state as well as overcoming technical barriers to linking data systems.

Although participants generally believed that patient data shared between facilities improved patient care, some also noted that the volume and disorder of data could create challenges for clinicians and case managers, who might require training to find, interpret, and apply this information effectively. Hospitals also needed personnel and infrastructure to analyze the data, which may have been easier for larger hospital systems.

### Global Budget Calibration

The HSCRC leveraged data, expertise, and relationships to negotiate budgets with individual hospitals, but study participants observed the persistent technical challenge of setting hospital global budgets. Regulators grappled with shifts in hospital volume, particularly for patients in urban settings or for hospitals seeking to expand or contract services. One state official noted, “That’s why global budgets are easier to implement in a rural setting. A lot of the complications are when you’re in an urban setting with volume moving around. You don’t want [hospitals] to just shift volumes around and keep money that doesn’t make sense [interview 9].” Special circumstances for hospitals also created challenges. These included capital investments, including new construction and information technology, as well as the high cost of novel therapies such as pharmaceuticals. Academic medical centers posed specific challenges for establishing budgets, including early adoption of novel therapies, patients seeking specialized care from other markets and outside the state, and balancing efficiency with educational priorities.

To address the complex challenge of setting budgets for individual hospitals, regulators relied on data as well as communication with stakeholders. In addition, Maryland established grant funding separate from the global budget specifically for innovation in priority areas including population health and behavioral health, with the long-term goal that these investments would be sustained within the global budget. A state regulator stated, “We gave them infrastructure money in the global budget for population health…it wasn’t necessarily being spent on things that we thought were population health oriented. So, we moved on to provide more specific grant programs where hospitals had to propose initiatives to get extra money in their budget [interview 11].”

### Shared Commitment to Change

An important facilitator for the MDAPM was that stakeholders shared motivation to adopt the new approach. Before implementation of the model, the waiver that had allowed Maryland to operate the rate-setting system was threatened owing to rising cost. The implications of losing this waiver and radical consequences of reverting to the systems present in other states exerted external pressure that united stakeholders. One state leader noted, “The way people thought: Do I want to spend the next 5 years chasing after revenue from nongovernment payers, or do I want to do the hard work of redesigning how care is delivered and do the right thing? It became a relatively easy choice [interviewer 1].” Some participants believed that these circumstances applied only to Maryland given its unique hospital rate-setting authority. However, participants highlighted other cultural and structural factors that helped to align stakeholders in response to this external threat, factors that were necessary for change but not specific to the state. One hospital leader commented, “I think it was not just the HSCRC, but also the concept of collaboration. That you could set a statewide goal, and everyone could work on it.... You were building on a base of trust. In other places, people are vicious competitors [interview 17].”

Another facilitator that strengthened commitment to change was that the model was not designed by the CMS alone but in close partnership with the state. In turn, the state sought to engage hospitals and payers early in the design. Participants commented that this collaboration customized the MDAPM to the specific state environment and also mitigated future implementation barriers.

## Discussion

In this study, we found common and interrelated themes in the perspectives of health care leaders on the development of hospital global budgets in Maryland. As value-based payment continues to evolve, hospital global budgets may be an important tool to drive both transformation of the health care system and more sustainable cost growth. Our findings point to strategies that may aid implementation of hospital global budgets in other settings as well as hazards inherent to this approach.

This study was not intended to evaluate the effectiveness of the MDAPM on cost or quality; rather, we sought to identify important lessons that emerged from model implementation. Each theme contains both barriers and facilitators to adopting hospital global budgets, which together offer lessons that can be extrapolated from the Maryland experience. For example, autonomy was important for hospitals, allowing each health system to develop its own initiatives in response to population and organizational needs. However, this autonomy might mean that some hospitals are slower to invest in quality improvement efforts. Overall, these interweaving barriers and facilitators portray model implementation as a dynamic process, requiring collaboration between stakeholders before implementation and in the years afterward. The themes identified in this study reflect elements necessary for learning, including close communication, accurate data to guide decisions, and perseverance stemming from collective commitment to change.

A common criticism of the MDAPM is that it is not generalizable beyond Maryland owing to the historical all-payer rate-setting system.^7,23^ The findings of this study suggest that this rate-setting system was important in that it provided technical infrastructure to implement the model and a shared commitment to payer-agnostic hospital reimbursement. However, the study participants noted that one reason for successful MDAPM implementation was that it was designed for the unique circumstances in the state in collaboration with stakeholders from its inception. A replica of the MDAPM might not be relevant for other states, but shared participation in payment model design is a lesson that can be applied broadly.

Previous studies of the MDAPM have debated its impact on cost and hospital utilization.^7^ The independent, federally funded evaluation of the model demonstrated savings in total cost and decreased admissions for Medicare as well as savings in total hospital expenditures for the commercially insured population.^3^ However, hospital global budgets may offer benefits beyond cost containment. They may be used to maintain access to emergency and acute care by preventing hospital closures in rural areas as well as for urban safety-net facilities.^12,13^ Also, some evidence demonstrates increased resiliency to loss of volume during the COVID-19 pandemic.^19^ In this study, participants noted that global budgets were not designed to severely constrain hospital spending but rather to incrementally realign hospital incentives, with additional quality measures that were perceived as useful to ensuring focus on improving patient outcomes.

Maryland has itself progressed to the Total Cost of Care Model, which seeks to improve on limitations of the MDAPM.^25^ These strategies include increased investment in primary care, strengthened alignment between physicians and hospitals, and an explicit focus on improved population health. The present study found that hospital global budgets, though a paradigm shift, were an intermediary step toward accountability for total cost. Strengthening incentives for hospitals to focus on community needs for both acute and preventive care is a key goal for Maryland, and the savings from reduced hospital growth are expected to allow for greater investment in primary care and population health. However, engaging health care professionals beyond the hospital remains a challenge that is necessary to achieve true system-wide transformation.^4^

### Limitations

This study has several limitations. First, the study used qualitative methods, and the results should therefore be interpreted as generating hypotheses for further evaluation. Second, all study participants were vested in the MDAPM and thus may be biased to interpret its outcomes positively, although many openly shared criticisms and weaknesses of the model. Third, this study was conducted 5 years after the MDAPM launch, allowing participants to reflect on relatively long-term outcomes but also creating the potential for recall bias. Fourth, data collection concluded just before the COVID-19 pandemic, which will have implications for future health system design. Finally, the study by design excluded some important perspectives on the topic, including those of patients.

## Conclusions

In this qualitative study, we interviewed health care leaders to identify generalizable lessons from the MDAPM, one of the most sweeping forays into value-based payment in the US. We identified 6 key themes: (1) expectations, (2) autonomy, (3) communication, (4) actionable data, (5) global budget calibration, and (6) shared commitment to change. These themes merit further examination and may be considered in the design, implementation, and analysis of other hospital global budget models.

## Notes

Supplement. **eFigure.** Map of Maryland All-Payer Model (MDAPM) Stakeholders **eMethods 1.** Interview Guide **eMethods 2.** Qualitative Codebook: List of Codes Used for Qualitative Content Analysis and Table of Definitions
