# Supplementary material for: Health Care Leaders’ Perspectives on the Maryland All-Payer Model
Source: JAMA Health Forum. 2022 Feb 4;3(2):e214920. doi: 10.1001/jamahealthforum.2021.4920 (PMC8903109; doi:10.1001/jamahealthforum.2021.4920)
Supplement: Supplement. — eFigure. Map of Maryland All-Payer Model (MDAPM) Stakeholders eMethods 1. Interview Guide eMethods 2. Qualitative Codebook: List of Codes Used for Qualitative Content Analysis and Table of Definitions [file jamahealthforum-e214920-s001.pdf]

Supplementary Online Content

Kilaru AS, Crider CR, Chiang J, Fassas E, Sapra KJ. Health care leaders' perspectives on the Maryland all-payer model. *JAMA Health Forum*. 2022;3(2):e214920. doi:10.1001/jamahealthforum.2021.4920

eFigure. Map of Maryland All-Payer Model (MDAPM) Stakeholders

eMethods 1. Interview Guide

eMethods 2. Qualitative Codebook: List of Codes Used for Qualitative Content Analysis and Table of Definitions

This supplementary material has been provided by the authors to give readers additional information about their work.

© 2021 Kilaru AS et al. *JAMA Health Forum.*

__eFigure. Map of Maryland All-Payer Model (MDAPM) stakeholders__

Red outline indicates stakeholders participating in this study; black outline indicates stakeholders excluded from this study. Blue shade indicates affiliation with hospitals, providers, and service organizations; yellow shade indicates affiliation with state government; green shade indicates affiliation with payer, including federal.

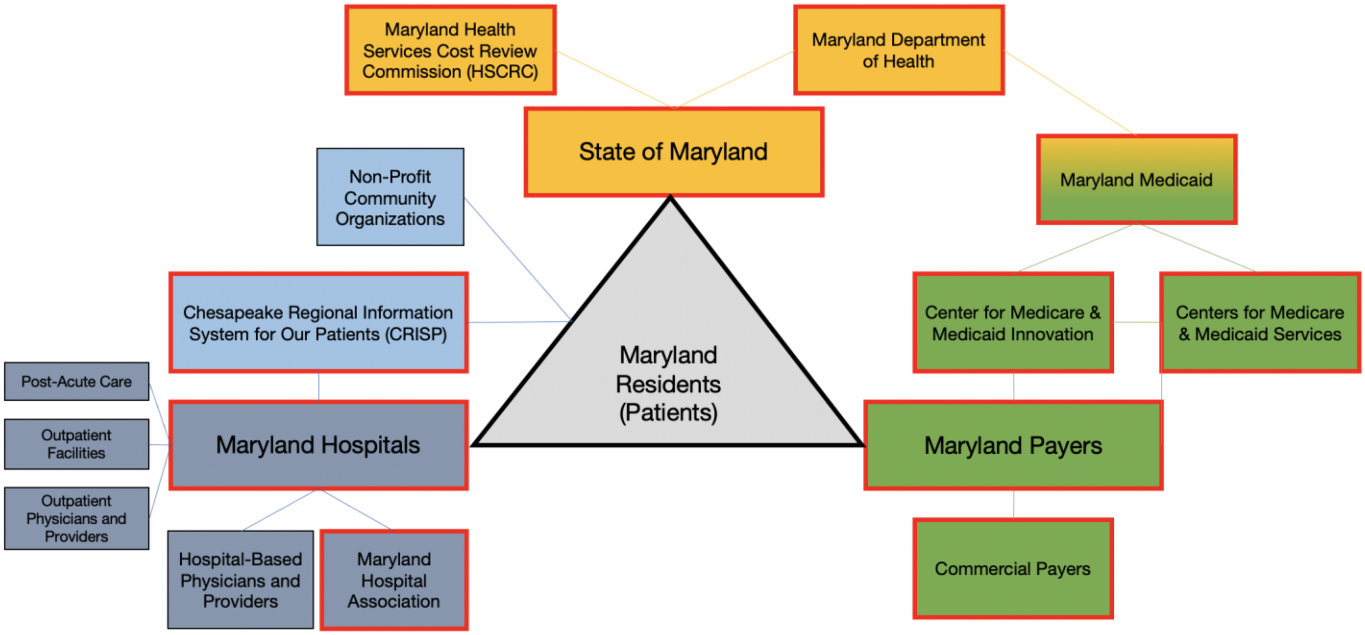

© 2021 Kilaru AS et al. *JAMA Health Forum.*

eMethods 1. Interview Guide

Background

1. **Please tell me about your role in the development of the Maryland APM.**

2. **How do you interpret outcomes of the global hospital budget phase of the Maryland APM?**

   Prompt
   – How is the model impacting patients and population health?

General

3. **What were the key barriers for adoption and implementation of the Maryland APM?**

4. **What were facilitators for adoption and implementation of the Maryland APM?**

Inner Setting

Implementation Climate

5. **Describe the readiness of stakeholders for implementation of the Maryland APM.**

   Prompts
   – What were the incentives in place for joining this model?
   – What did hospitals expect to gain or lose from participating?
   – What were the goals of the state of Maryland?

6. **What were your priorities during the design and implementation of the Maryland APM?**

Structural Characteristics

7. **How did the structure of the healthcare system in Maryland allow for the development of this model?**

   – What aspects of the culture within Maryland contributed to development of the model?
   – How did the political environment contribute to adoption of the Maryland APM?
   – Describe the relationships between key stakeholders prior to implementation of the model.

Outer Setting

External policies and incentives

8. **What external forces or pressures on Maryland influenced development of the APM?**

   Prompt
   – What were alternatives for stakeholders to the APM model?

Individual Characteristics

9. **From your perspective, who were the key individual or institutional leaders for the APM?**

© 2021 Kilaru AS et al. *JAMA Health Forum.*

Process

Planning

10. **From your perspective, what were the pivotal decisions in planning for the APM?**

    Prompt
    – How were goals for the Maryland APM set, accepted, and perceived?

Reflection and Evaluation

11. **How did stakeholders reflect, evaluate, and provide feedback as the model was launched?**

    Prompt
    – What changes had to be made to the model after its initial design and implementation?

Intervention Characteristics

12. **From your perspective, what were the key features of the design of the Maryland APM model design that led to its outcomes?**

    Prompt
    – What were the key components of financial arrangements in the APM model?

13. **What barriers were encountered during implementation of the APM, and how were they addressed?**

    Prompts
    – What was the perceived complexity of the model and its requirements?
    – What burdens did the model place on hospitals and other stakeholders?
    – What were unforeseen difficulties or barriers that were recognized after implementation?

14. **What were key facilitators for the outcomes of the APM?**

    Prompts
    – What was the role of data and information exchange?
    – How did communication and collaboration between entities facilitate implementation?

Conclusions

15. **What key aspects of the experience implementing the Maryland All-Payer model should be extended to other settings?**

© 2021 Kilaru AS et al. *JAMA Health Forum.*

eMethods 2. Qualitative Codebook: List of Codes Used for Qualitative Content Analysis and Table of Definitions

General codes

- **Barriers to implementation**
- **Facilitators of implementation**
- **Outcomes and consequences**
- **Key decisions and processes**

Topic/thematic codes

- **Administration**: necessary operational responsibilities, actions, and challenges
- **Alignment:** hospital and provider alignment
- **Communication:** communication and collaboration between state, federal government, and stakeholders
- **Data:** collection and use of data for performance measurement
- **Flexibility:** adjustments and amendments to model over time
- **Model:** technical and financial aspects of model design
- **Past:** prior Maryland systems, policies, experience
- **Patients**: effects on patients and population health
- **People:** leaders and influencers
- **Setting**: effect of environmental context on implementation (eg urban or rural settings)
- **Strategies**: hospital strategies, competition, and innovation
- **Sustainability**: what does this approach seek to achieve over the long-term, and how can it stay viable
- **Transition:** system transition and change management

© 2021 Kilaru AS et al. *JAMA Health Forum.*

Code	Definition, key questions, and notes	Related Codes
General codes		
**Barriers to implementation**	- Key obstacles that caused resistance and uncertainty in planning, adopting, and executing the MDAPM	Key decisions
**Facilitators to implementation**	- Key features of the MDAPM that helped its planning, adoption, and execution in Maryland	Key decisions
**Outcomes and consequences**	- Positive or negative changes did this model generate for all stakeholders – patients, providers, hospitals, regulators, spending<br>o Intended consequences<br>o Unintended consequences<br>o Short-term effects<br>o Long-term effects	Hospital strategies
**Key decisions and processes**	- Key decisions and choices that were made in the design and implementation of the MDAPM<br>- Key processes or steps that had to occur for implementation	Barriers; facilitators
Topical Codes		
**Administration**<br>Operational responsibilities, actions, and challenges needed to respond to the MDAPM	- Necessary operational actions and responsibilities for stakeholders and hospitals as a result of the MDAPM<br>o Challenges posed by administrative aspects of the model<br>o Personnel and recruitment issues?<br>o Contracting and other logistical and financial tasks	Hospital strategies; Barriers; Alignment
**Alignment**<br>hospital and provider alignment	- Alignment of providers with hospitals in the MDAPM<br>- Hospitals strategies to establish alignment with providers, including physicians as well as non-hospital care settings?	Hospital strategies

© 2021 Kilaru AS et al. *JAMA Health Forum.*

**Communication**<br><br>communication and collaboration between state, federal government, and stakeholders	- Stakeholders collaboration to produce and execute the model<br>o Information communicated among stakeholders<br>o Facilitators and barriers to collaboration and communication	Transition; leaders; facilitators
**Data**<br><br>collection and use of data for performance measurement	- Collection and deployment of data in the execution of the MDAPM<br>o How did stakeholders use data?<br>o What was the role of data in the outcomes of the MDAPM?<br>o Was data useful for hospitals or challenging to use?	Facilitators; hospital strategies
**Flexibility**<br><br>adjustments and amendments to the model over time	- Changes the model over time<br>o What changes were needed by stakeholders over time?<br>o How receptive were regulators to suggested changes?<br>o How effective were changes made over time?	Communication
**Model**<br><br>technical and financial aspects of model design	- Important technical details of the model design<br>o What was challenging about crafting the technical details of this model?<br>o What were key financial aspects or design elements of the model that were perceived to be important?	Data; Flexibility
**Past**<br><br>Prior Maryland systems, policies, experience	- Aspects of the previously existing system in Maryland influencing implementation and outcomes	Facilitators
**Patients**<br><br>effects on patients and population health	- Patient experience of the MDAPM<br>- Goals and effects on population health<br>o How did different populations of patients experience the MDAPM?<br>o What challenges did patients pose for hospitals in the MDAPM?	Good outcomes; bad outcomes; hospital strategies
**People**<br><br>leaders and influencers	- Leaders, first-adopters, champions, and influencers that promoted implementation and change?<br>o How did they lead or influence changes?	Facilitators
**Setting**<br><br>effect of geography and community context on implementation	- Effect of geography, environmental context or other external circumstances on the model<br>o How did the effects or implementation of the MDAPM vary across the state?<br>o What was the effect in urban settings versus rural settings?	Positive outcomes; negative outcomes

© 2021 Kilaru AS et al. *JAMA Health Forum.*

**Strategies**<br><br>hospital strategies, competition, and innovation to proactively capture new opportunities presented by model.	- What strategies were effective for hospitals?<br>- What changes did the program create for hospitals?<br><br>o What programs did hospitals start to meet model requirements?<br>o To what extent were hospitals able to innovate under the MDAPM?<br>o How did hospitals compete for market share under the MDAPM?<br>o Inpatient versus outpatient spaces<br>o Regulated versus unregulated spaces	Data performance, hospital and provider alignment; goals; transition; administrative challenges
**Sustainability**<br><br>long-term success and participation in the model	- Design of the model with intent of long-term sustainability<br><br>o What is needed to maintain Model viability?<br>o What does this approach seek to achieve over the long-term?	Positive outcomes, Key decisions
**Transition**<br><br>system transition and change management techniques undertaken by hospitals, providers, and the state	- Adjustments that hospitals and other stakeholders had to make during transition from fee-for-service to hospital global budget<br><br>o Change management strategies used by stakeholders	Barriers; facilitators; hospital strategies

© 2021 Kilaru AS et al. *JAMA Health Forum.*
